# Supplementary material for: Quasi-Freeform Metasurfaces for Wide-Angle Beam Deflecting and Splitting
Source: Nanomaterials (Basel). 2023 Mar 24;13(7):1156. doi: 10.3390/nano13071156 (PMC10097112; doi:10.3390/nano13071156)
Supplement: Supplementary file 1 [file nanomaterials-13-01156-s001.zip › nanomaterials-2274456-supplementary.pdf]

nanomaterials

MDPI

Supporting information

Quasi-freeform metasurfaces for wide-angle beam deflecting and splitting

Qiuyu Zhang [1,2,3], Dingquan Liu[1,2,3,*], Sheng Zhou[1], Gang Chen[1], Junli Su[1,2],Leihao Sun[1,2], Yunbo Xiong[1,2], and Xingyu Li[1,2]

[1] Shanghai Institute of Technical Physics, Chinese Academy of Sciences, Shanghai 200083, China
[2] School of Physical Science and Technology, ShanghaiTech University, Shanghai 200031, China
[3] School of Optoelectronics, University of Chinese Academy of Sciences, Beijing 100049, China
* Correspondence: dqliu@mail.sitp.ac.cn; Tel.: +86-21-25051303; Mobil: +86 15216701692

1. Polarization dependence discussion

Calculated total splitting efficiency and splitting uniformity of optimized supercells for three-port splitting output under y polarized light are depicted in Figure S1. Clearly, the results are not good as the simulation values in Figure 4b, illustrating that the proposed metasurfaces are polarization dependent.

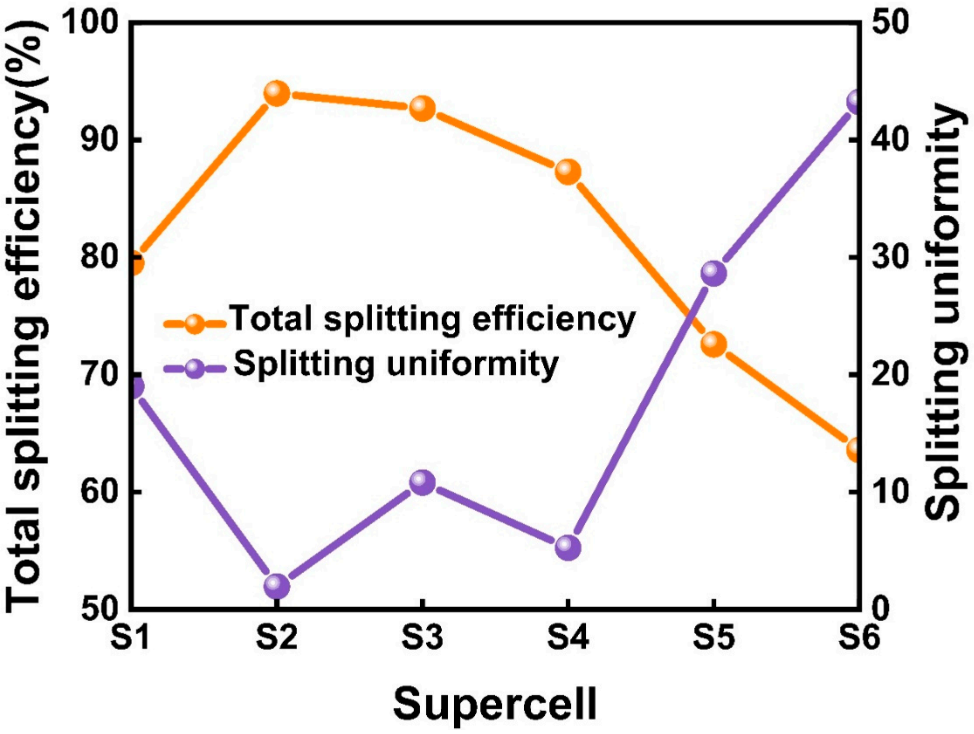

Supplementary Figure S1. Splitting efficiency and splitting uniformity of the optimized supercells under y polarized light.

2. Structure parameters for optimized supercells

We have summarized the structure parameters of optimized supercells in Table S1. The radius and location of the nanocylinders in supercell are represented by a one-dimensional matrix.

Supplementary Table S1. The structure parameters for optimized supercells

Supercell	Radius(nm)	Location(nm)	Height(nm)
**D1**	[153, 176, 190, 208]	[-866, -229, 387, 881]	759
**D2**	[159, 180, 203]	[-512, 124, 677]	764
**D3**	[163, 185, 222]	[-393, 63, 550]	700
**D4**	[187, 163]	[-751, 576]	821
**D5**	[204, 162]	[-714, 503]	819
**D6**	[159, 198]	[-216, 222]	787
**S1**	[63, 163, 164, 66]	[-865, -162, 372, 847]	738
**S2**	[110, 161, 164]	[-1076, 200, 733]	800
**S3**	[117, 160, 158]	[-391, 267, 691]	783
**S4**	[165, 61]	[-24, 769]	755
**S5**	[179, 143]	[-442, 404]	713
**S6**	[181, 148]	[-437, 362]	713
**S7**	[184, 138, 94, 142]	[-1097, -255, 338,1114]	743
**S8**	[242, 125,124, 125, 242]	[-1336, -858,0, -858, -1336]	552
**S9**	[195, 141,185, 141, 195]	[-1960, -743,0, -743, -1960]	762
